# Natural and Unanticipated Modifiers of RNAi Activity in *Caenorhabditis elegans*

**DOI:** 10.1371/journal.pone.0050191

**Published:** 2012-11-28

**Authors:** Nadeem Asad, Wen Yih Aw, Lisa Timmons

**Affiliations:** 1 Department of Molecular Biosciences, The University of Kansas, Lawrence, Kansas, United States of America; 2 Undergraduate Honors Program, The University of Kansas, Lawrence, Kansas, United States of America; Iowa State University, United States of America

## Abstract

Organisms used as model genomics systems are maintained as isogenic strains, yet evidence of sequence differences between independently maintained wild-type stocks has been substantiated by whole-genome resequencing data and strain-specific phenotypes. Sequence differences may arise from replication errors, transposon mobilization, meiotic gene conversion, or environmental or chemical assault on the genome. Low frequency alleles or mutations with modest effects on phenotypes can contribute to natural variation, and it has proven possible for such sequences to become fixed by adapted evolutionary enrichment and identified by resequencing. Our objective was to identify and analyze single locus genetic defects leading to RNAi resistance in isogenic strains of *Caenorhabditis elegans*. In so doing, we uncovered a mutation that arose *de novo* in an existing strain, which initially frustrated our phenotypic analysis. We also report experimental, environmental, and genetic conditions that can complicate phenotypic analysis of RNAi pathway defects. These observations highlight the potential for unanticipated mutations, coupled with genetic and environmental phenomena, to enhance or suppress the effects of known mutations and cause variation between wild-type strains.

## Introduction

RNA silencing is a term commonly used to describe the multiple, overlapping, RNA-directed pathways that are used by cells to regulate gene expression and chromatin functions. RNA silencing mechanisms serve as cellular sentinels, providing fundamental anti-foreign genome defense activities that protect cells against transposon mobilization and organisms against systemic viral infection. RNA silencing pathways include RNA interference (RNAi) mechanisms that are triggered when dsRNA is provided to cells. Hundreds of genes have been implicated in RNA silencing using forward genetic and RNAi-based screens, which is a testament to the scope of these mechanisms in cells. Genetic defects in some members of these pathways can lead to developmental or environmentally-influenced phenotypes in *Caenorhabditis elegans*. Considering the vital and fundamental nature of RNA silencing functions, it is surprising that relatively few RNA silencing genes are required for viability or fertility, suggesting a functional overlap of RNA silencing activities.

One practical consequence of the interwoven nature of RNA silencing networks is that mutations in these pathways are often tolerated by the organism. Indeed, several unexpected mutations that affect RNA silencing have been identified in laboratory strains as well as in feral isolates [1], [2]. The mutations found in laboratory strains are considered background mutations as the ancestry of the mutations can often be traced back to a common source. The lack of a selective disadvantage in the laboratory environment may contribute to the perpetuation of such mutations, especially considering that *C. elegans* strains are normally cultivated in the laboratory in a manner that does not typically select for animals with “wild-type” levels of RNA silencing activity. Alternatively, a selective advantage under some genetic or environmental conditions might contribute to fixation of RNA silencing mutations.

During the course of our studies on RNA silencing mechanisms, we observed that some of our strains harbored unanticipated mutations that affect RNAi. Unlike previously reported background mutations, these have a more recent genesis. Here we report novel mutations in genes that encode Dicer-interacting proteins, as well as long-lasting maternal effects, that allow for RNAi activity in adults. The recurrent observations of background mutations affecting RNA silencing mechanisms, as well as environmental influences and maternal effects that can influence the robustness of RNAi and thereby obscure underlying RNAi-related mutations, highlights the importance of comparing multiple alleles or differently outcrossed strains, as such unexpected mutations may have pleiotropic effects that could complicate phenotypic analyses.

## Materials and Methods

### C. elegans strains

*Mapping and other strains (from A. Fire):* PD2119 [*dpy-1*(*e1*) *ncl-1*(*e1865*) III], PD2039 [*dpy-5*(*e61*) *unc-54*(*e1092*) I, PD2014 [*dpy-10*(*e128*) *unc-52*(*e669*)] II, PD2027 [*unc-17*(*e245*) *dpy-4*(*e1166*)] IV, PD9064 [*dpy-11*(*e224*) *unc-60*(*e723*)] V: pPD4300 [ccIs4251 (myo-3::GFP) I; ayIs2 (egl-15::GFP) IV; ayIs6 (hlh-8::GFP) X]; pPD5063 [ccIs4251 (myo-3::GFP) I]; PD8175 [*rde-1(ne219)*].

*Deletion strains used to map rde-4(ne309) (from the* Caenorhabditis *Genetics Center):* BC4637 [sDf130(*s2427*) *unc-32*(*e189*) III; sDp3 (III;f)], TY1353 [yDf10 *unc-32*(*e189*)/*unc-93*(*e1500*) *dpy-17*(*e164*) III], BC4638 [sDf127(*s2428*) *dpy-17*(*e164*) *unc-32*(*e189*)III; sDp3 (III;f)], BC4697 [sDf121(s2098) *unc-32*(*e189*) III; sDp3 (III;f)], MT1928 [nDf16/*unc-36*(*e251*) *dpy-19*(*e1259*) III], MT5491 [nDf40 *dpy-18*(*e364*) III/eT1 (III;V)], DG801 [*unc*-32(*e189*) tnDf2/*sma-2*(*e502*) *ced-7*(*n1892*) *unc-69*(*e587*) III], CB4118 [*unc-32*(*e189*) *ooc-4*(*e2078*)/eDf20 III], GE2158 [tDf2/qC1 *dpy-19*(*e1259*) *glp-1*(*q339*) III], GE2180 [*unc-32*(*e189*) tDf7/qC1 *dpy-19*(*e1259*) *glp-1*(*q339*) III; *him-3*(*e1147*) IV], BW1535 [*dpy-18*(*e364*) ctDf3 *unc-25*(*e156*)/qC1 *dpy-19*(*e1259*) *glp-1*(*q339*) III], BW1369 [*unc-32*(*e189*) *dpy-18*(*e364*) ctDf2/qC1 *dpy-19*(*e1259*) *glp-1*(*q339*) III], NG2618 [yDf10 *unc-32*(*e189*)/qC1 *dpy-19*(*e1259*) *glp-1*(*q339*) III], CX2914 [nDf16/*dpy-17*(*e164*) *unc-32*(*e189*)III]

*Other strains (from the* Caenorhabditis *Genetics Center):* CB879 [*him-1*(*e879*)], AZ244 [*unc-119*(*ed3*)III; ruIs57], BB1 [*dcr-1*(*ok247*)/*unc-32*(*e189*) III], NL1820 [*mut-7*(*pk720*) III]

*New strains:* XX636 [*rde-4*(*ne309); him-1*(*e879*) I], XX637 [*dpy-1*(*e1*) *unc-119*(*ed3*) III], XX47 [*rde-4*(*ne309*)], XX1849 [*him-1*(*e879*) I; *rde-4*(*ne309*) III], XX1076 [*ccIsPD4251*(*myo-3::GFP)*] I; *yyEx1.1*(*myo-3::gfp hairpin, rol-6(su1006)*)]; XX528 [*haf-6(ne335) ccIsPD4251*(*myo-3::GFP*)] I; *yyEx1.1*(*myo-3::gfp hairpin, rol-6(su1006)*)], XX1151 [*ccIs4251(myo-3:: GFP)* I; *him-8(me4)*IV; *rde-1(yy11)* V, XX1278 [*ccIs4251(myo-3:: GFP)* I; *him-8(me4)*IV; *rde-1(yy11)* V; XX1835 [*rde-4*(*ne299*); *ccIs4251*(*myo3::GFP*); *yyEx1.1*(*myo-3 promoter::gfp hairpin* ; *rol-6*(*su1006*)], XX1823 [*him-5*(*e1490*) V; ccIs4251(*myo3 promoter::GFP*)]; Strains XX103, XX172, XX183, XX193, XX194, XX195, XX364, and XX526 are RNAi defective strains that are homozygous for the *haf-6(ne335)* mutation.

*Transgenic strains:* The ccIs4251 and yyEx1.1 transgenes used here have been described [3], [4]. The two transgenes were introduced together in wild-type and mutant backgrounds using standard genetics. Both transgenes utilize a *myo-3* promoter, which is transcriptionally active in muscle from late embryogenesis through adulthood. ccIs4251 expresses a GFP reporter, and was obtained by inducing an extrachromosomal array to integrate into the middle of chromosome I near the *rde-2* gene [3]. The yyEx1.1 transgene contained a *myo-3* promoter that drives *gfp* dsRNA expression in muscle from repetitive extrachromosomal DNA sequences that were assembled by the worm from injected plasmids. The ccIs4251 transgene allows for muscle-specific accumulation of *gfp* mRNA and of GFP reporter protein and is the target of RNAi in this double transgene RNAi system.

### Plasmids

The pLT494 and pLT495 plasmids contain *rde-1*(*yy11*) genomic region defined by primers 164 and 207 subcloned into pCR2.1 for sequencing. Amplification of this transposon insertion allele typically produced ∼2300 bp and ∼700 bp PCR products, respectively, presumably due to slipped strand mispairing caused by the Tc1 inverted repeats.

### Primers

*rde-1 primers:* 164: ggaagaaatgcaaaaaagtac; 165: ttagggtattttctttgtag;

170: tatcaatccaggtggaactat; 171: ttcatatcctcctcccattgtt; 207: atcccgttccgacatgaattg; 717: atatatatgtcctcgaattttcccga; 718: atatattgcgaacgacattccagg;

1142: gatctctctagctggccaccaaacatt; 1143: gtttcctgtaaccaaaatatagtcttc;

1144: ggtatttagtgtacaatttttgtagaa; 1145: caccgaactaattggtggttgcaagtt;

1249: gcgatcacaccatcggtgtagctaa; 1250: tcaccagaagaaaaagaaagacgga

*Tc1 primers:* 1333: tcgataatcatgtaatgtttggtc; 1334: atccatttgacttgaatttttccgt;

1335: gaaacttcaccacagtgttcacaaaatct; 1336: ttcgtattgaaaacgatccataatgcttt;

1337: gacaacccataggatggatcgcaacatcc

*rde-4 primers:* 209: gaaggatccatggatttaaccaaactaacg; 719: atatatatccgtgaaatcataggtg

### PCR- based detection of *rde-1(yy-11)* allele in mixed populations

Animals were washed with water and suspended in NET buffer (100 mM NaCl, 20 mM EDTA, 50 mM Tris-HCl, pH8.0) and frozen. 1∶200 volume of 10% SDS and 1 ug proteinase K were added to thawed pellets, which were incubated at 65°C for 2–4 hours, then phenol extracted, and ethanol precipitated. Primer set 1249/207 anneals to the *rde-1* gene, producing an amplified product of 640 bp in wild-type animals. (This primer set preferentially amplifies the wild-type allele in mixed populations of *rde-1(yy11)* and wild-type animals.) Primer set 1249/1333 specifically amplifies the *rde-1(yy11)* insertion, yielding an amplification product of 279 bp.

### dsRNA delivery by “feeding”

Standard *C. elegans* plates supplemented with cholesterol, ampicillin, tetracycline, and IPTG were seeded with the *Escherichia coli* strain HT115(DE3) harboring feeding plasmids as described [5], [6], [7], [8]. Transformed bacteria harboring *pop-1* cDNA in L4440 vector transcribe dsRNA that targets the TCF/LEF1 transcription factor, inducing sterility in young animals reared on this food, while the *dpy-11* food targets a protein that affects body shape, eliciting a shorter and fatter appearance in comparison to wild type. Control experiments utilized wild-type worms and empty L4440 vectors. Experiments were performed at 15°C, 20°C and 25°C.

### Genetic analysis of *RDE-4* maternal effects

To assess the extent of RDE-4 maternal effects, we first generated strains such as XX1835, which harbors the *rde-4(299)* mutation, the yyEx1.1 *gfp* dsRNA-expressing extra-chromosomal array, and the integrated ccIs4251 GFP reporter. Homozygous *rde-4* animals (XX1835, as well as a similar strain harboring the *rde-4(ne309)* mutation) were mated with XX1823 males, which harbor the GFP reporter. F1 progeny harboring the yyEx1.1 *gfp* dsRNA-expressing transgene array marked with a dominant “roller” transformation marker, as well as the GFP reporter, were individually placed on standard *C. elegans* culture plates. F2 animals harboring the “roller” phenotype were scored for RNAi activity as young adults and subsequently genotyped by DNA sequencing of the *rde-4* gene. 25% of such F2 progeny are predicted to be homozygous for the *rde-4* mutation. Before sequence analysis, some F2 adults were again placed as individuals on standard culture plates and allowed to produce F3 animals. The transgenic F3 animals were scored for RNAi activity and some were also sacrificed for DNA sequencing. 25% of the plates with such F3 progeny are predicted to be homozygous for the *rde-4* mutation. Animals were tracked, and the genotype was correlated with phenotype at the end of each experiment. We scored animals as RNAi+ if they displayed GFP fluorescence in fewer than 30% of muscle cells and RNAi- when GFP fluorescence in was observed in 70–90% of muscle cells. (All RNAi- animals in these assays displayed bright GFP fluorescence in >90% of muscle cells.) Experiments were conducted at 25°C, 22°C and 15°C.

## Results and Discussion

### A novel allele of *rde-1* arose spontaneously in a transgenic stock

**Figure 1:**
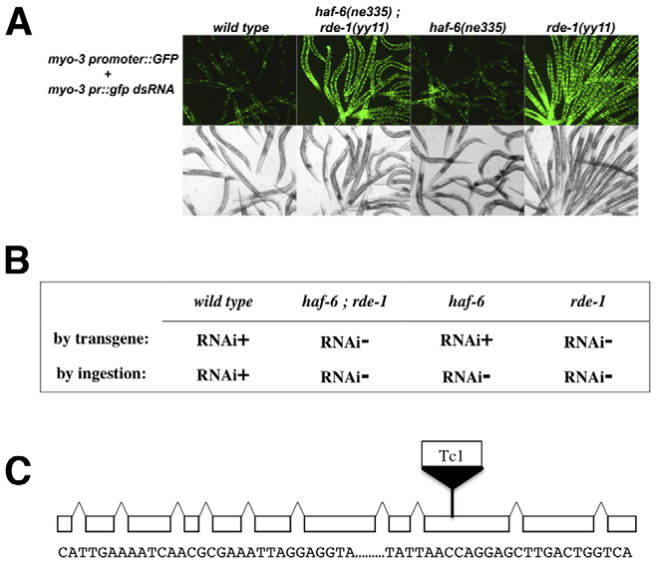
RNAi activity in *rde-1(yy11)* and *haf-6(ne335)* mutants. The *yy11* allele, present as a background mutation in a *haf-6(ne335)* transgenic stock, was genetically dissected from the *haf-6(ne335)* mutation and analyzed separately for RNAi activity. (**A**) *yy11* provides resistance to transgene-delivered dsRNAs, as evidenced by lack of gene silencing of the GFP reporter in homozygous animals. By contrast, *haf-6(ne335)* single mutants display wild-type RNAi activity in this transgene assay. (**B**). Animals homozygous for the *yy11* allele are RNAi resistant when dsRNAs are delivered by ingestion of bacteria that express dsRNAs. Thus, the *rde-1(yy11)* mutants displayed strong RNAi phenotypes irrespective of delivery method (**A**, **B**), while, as expected, the *haf-6(ne335)* single mutant was RNAi defective by ingestion only (**A, B**). Bacteria expressing dsRNA targeting the *pop-1* and *dpy-11* genes were used in feeding experiments. (**C**) Genetic and molecular analysis revealed the nature of the *yy11* mutation—a transposon insertion in exon 9 of *rde-1.* DNA sequences flanking the insertion site are indicated.

As an extension of experiments designed to uncover roles for the *Caenorhabditis elegans* ABC transporter gene *haf-6* in RNA interference [9], [10], we built strains to determine if *haf-6* is required for RNAi when dsRNA is transcribed from transgenes. The strains harbored two distinct configurations of transgenic DNA such that *gfp* sequences served as both trigger and target of RNAi, allowing for direct visual inspection of RNAi activity. In a wild-type background, this dual transgene system allows for easy visualization of an RNAi response against *gfp*. Our experiments using these transgenic strains led to observations of RNAi defects (Figure 1). The RNAi defects were not rescued by the introduction of wild-type *haf-*6 sequences, and we ascertained that the transgene-mediated RNAi defects were due to an unknown mutation, which we termed *yy11,* present in the background of the transgenic *haf-6(ne335)* strain.

In order to identify the nature of the gene with the *yy11* mutation, we employed standard genetic methodology to separate the recessive *haf-6(ne335)* and *yy11* mutations (Figure 1A,B). The resulting single mutant *yy11* homozygotes displayed RNAi defects in response to transgene-delivered dsRNAs, as well as defects in response to ingestion of dsRNAs targeting genes expressed in the germ line or soma. By contrast, the *haf-6(ne335)* single mutants were RNAi-defective in response to ingested dsRNA, as expected, but did not display RNAi defects in response to transgenes. Thus, the transgene-mediated RNAi defect was solely due to the unknown *yy11* mutation.

We mapped the *yy11* mutation to chromosome V. Considering that the *yy11* mutation elicits a strong RNAi defect, we reasoned that the corresponding gene might already have been uncovered in screens for RNAi-defective mutants. We therefore performed complementation tests between *yy11* strains and RNAi-defective worms harboring mutations in genes located on chromosome V. The *rde-1(ne219)* mutation failed to complement the RNAi defect in *yy11,* suggesting that *yy11* is an allele of *rde-1.* We then sequenced the *rde-1* locus in our strain and found a novel Tc1 transposon inserted in exon 9 (Figure 1C). Tc1 elements are members of the large *mariner* superfamily of simple transposable elements that harbor a single transposase gene flanked by inverted repeats [11]. The *rde-1(yy11)* allele is a novel and unusual mutation in that most of the documented alleles of *rde-1* are single base substitutions.

We reasoned that the sequence of the Tc1 transposon insertion in *rde-1(yy11)* might help illuminate how the mutation arose in our lab. The *rde-1(yy11)* Tc1 element has almost 100% identity with a Tc1 element that resides within an intron of T07D3.3, a gene on chromosome 2. This sequence identity provides an indication as to the source of mobilized DNA, while the presence of a T insertion after base 204 in the *rde-1(yy11)* element (the only sequence difference between the two transposons) may reflect the recent nature of the transposition event. A potentially active transposon in *rde-1,* coupled with the ability to select for *rde-1* activity using RNAi methodology, raises the possibility of using the *rde-1(yy11)* allele as a screening or assessment tool for transposon silencing or RNAi activities.

**Figure 2:**
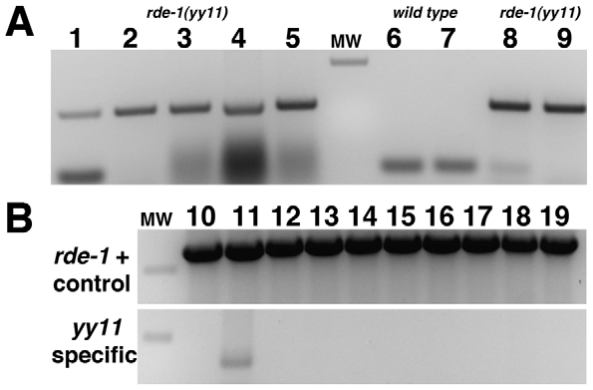
Origin of *rde-1(yy11)* mutation. A PCR-based assay was developed in order to detect the presence of the *rde-1(yy11)* allele in a mixed population of animals. (**A**) A single *rde-1(yy11)* homozygous animal was introduced into a tube with a number of hand picked, wild type adults. PCR reactions were performed using *rde-1(yy11)* allele-specific primers (1249/1333, *see* Materials and Methods). The ratios of *rde-1(yy11): wild-type* animals in each PCR assay were 1∶10 (lane 1), 1∶100 (lane 2), 1∶500 (lane 3), 1∶1000 (lane 4), and 1∶5000 (lane 5). The PCR-based assay was also performed on individual wild-type (lane 6) and *rde-1(yy11)* (lane 8) worms, as well as genomic DNA isolated from mixed-stage worms harvested from twenty-five 10 cm plates of mixed stage worms of wild-type (lane 7) or *rde-1(yy11)* (lane 9) genotype. (**B**) Frozen, archived stocks of mixed stage worms were thawed and sacrificed for genomic DNA isolation in order to determine the possible origin of the *rde-1(yy11)* allele. Each lane represents a PCR reaction from a different stock; for each strain the oldest tube from the archive was selected. The strains assayed harbored *haf-6(ne335)* alleles (lanes 11–18) or the ccIS4251 transgene insertion (lanes 10 and 19). Top panel in **B**: control PCR reaction using primers designed to amplify wild-type *rde-1* alleles (640 bp product, *see* Materials and Methods). Bottom panel: PCR reaction using primers specific for the *rde-1(yy11)* insertion allele (279 bp product, *see* Materials and Methods). The MW marker is 500 bp in size. The XX103 stock analyzed in lane 11 is an archived stock of the original, un-outcrossed *haf-6(ne335)* mutant stock; the other *haf-6(ne335)* stocks analyzed (lanes 12–18) are outcrossed or derivative strains.

The *rde-1(yy11)* allele was attained serendipitously–it was first observed in a *haf-6(ne335); rde-1(yy11)* double mutant (Figure 1). Two of the ancestors of this stock had been exposed to mutagens, which may have resulted in the transposition event that gave rise to the *rde-1(yy11)* allele [12]. One of these ancestral strains was the original *haf-6(ne335)* isolate; a second ancestral strain was exposed to mutagen in order to induce integration of the *ccIs4251* GFP reporter sequence [3]. We therefore hypothesized that the transposition event leading to the *rde-1(yy11)* mutation might have originated in such ancestral strains and thereafter lingered as a low frequency mutation. We designed a PCR-based assay to test for the presence of the Tc1 insertion using PCR and amplification conditions that would allow for the detection of the *rde-1(yy11)* allele in a mixed population of worms (Figure 2A). We applied this assay to progenitor strains, and strains later derived from them, for the presence of the *rde-1(yy11)* allele. We detected the allele in a frozen archived stock from the original, un-outcrossed *haf-6(ne335)* isolate (Figure 2B). *haf-6* mutants display mutator activity [10]; therefore, the *rde-1(yy11)* insertion may be a reflection of the transposon silencing defect in *haf-6* mutants.

The *rde-1(yy11)* mutation was present at low frequency in the background of the original *haf-6(ne335)* strain, as we did not detect the *yy11* allele PCR assays on smaller numbers of animals from this stock nor from genomic DNA isolated from all worms in similar tubes of archived stocks of various *haf-6* strains (our unpublished observations). Thus, even though the *haf-6(ne335)* strain used to build the transgenic strain in Figure 1A had been out-crossed to wild-type animals, the *rde-1(yy11)* mutation was not eliminated from the population. It is likely that the strategy we used to produce the transgenic *haf-6* strains resulted in the selected enrichment of *rde-1(yy11).* A recombination event was required to place the integrated *ccIs4251* GFP reporter transgene onto the same chromatid as *haf-6* (both elements reside in chromosome I). To facilitate the identification of the infrequent GFP-expressing *haf-6* recombinants, populations of progeny animals were selected for RNAi defectiveness using dsRNA-expressing bacteria, a strategy which may have contributed to the selection of the strong RNAi-defective *rde-1(yy11)* background mutation. It is interesting that GFP/*haf-6* recombinant animals that arose from this selection were also homozygous for *rde-1(yy11)*. We have not detected the *rde-1(yy11)* allele in other strains or in other batches of the same archived strain depicted in Figure 2; thus, this allele was eliminated from subsequent derivative strains, or lost from the population before the derivative stocks were frozen.

The novel *rde-1(yy11)* allele should prove to be a useful tool, facilitating analysis of the RNAi pathway in *C. elegans.* For example, an insertion allele such as *rde-1(yy11)* affords particular molecular advantages over existing *rde-1* alleles, all of which are single base substitutions, in that the insertion can be identified quickly using PCR. This strategy should allow for more expeditious molecular screening for *rde-1* mutations in situations where multiple mutations in the RNAi pathway must be brought together in the same strain. Additionally, the transposon insertion helps to confirm the null phenotype, allowing for a more complete phenotypic analysis. Existing *rde-1* alleles with experimentally established RNAi defects harbor small alterations to the DNA and/or protein sequence. These alleles are amino acid substitutions or disruptions that are downstream of the *yy11* insertion: for example, a E414K substitution in the *rde-1(ne219)* allele; G485E in *rde-1(ne4086);* I562L in *rde-1(ne4085);* G1016E in *rde-1(ne297);* and a Q825STOP mutation in *rde-1(ne300).* The Tc1 insertion in *yy11* occurs after amino acid G685, and is a more severe disruption than existing point mutations, meaning that it is also likely to be a null mutation.

### Maternal effects can mask the presence of mutations in genes with RNAi function

**Figure 3:**
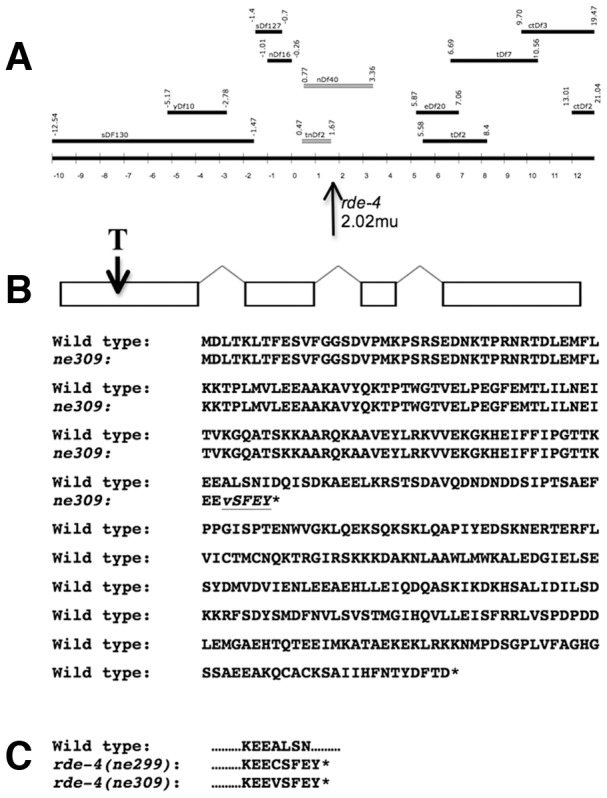
Genetic and molecular analysis of *rde-4(ne309)*. (**A**) Strains with the indicated chromosomal deficiencies were used in complementation tests in order to localize the *ne309* mutation. The deleted regions highlighted in gray failed to complement *ne309,* and the relative location of breakpoints is indicated on the genetic map. Breakpoint information is collected by the *C. elegans* community. The data reflects the information posted on Wormbase, version WS231. (**B**) The RNAi defect in *ne309* mutants is due to a single base insertion of a T residue in the first exon of *rde-4*. This mutation is predicted to lead to a truncated protein lacking the second of two double-stranded binding domains. The first dsRNA binding domain alone is unable to bind dsRNA [28]. (**C**) A similar T insertion in *rde-4(ne299)* affects the same codon.

We previously described a strain with an RNAi-defective allele, *ne309,* that does not mount an RNAi response to ingested dsRNAs [4]. We mapped the *ne309* mutation to chromosome III, and we performed complementation tests using large deletions to localize the *ne309* mutation and to help confirm that the strain harbored a single RNAi-defective allele (Figure 3A). Deletions lacking DNA sequences near the center of chromosome III failed to complement *ne309,* which led us to perform additional complementation tests between *ne309* and RNAi-defective strains with mutations in specific genes that map to this region. Among these genes was *rde-4,* which failed to complement *ne309.* Upon sequencing of the *rde-4* gene in *ne309* mutants, we found a mutation consisting of a single T insertion in exon 1. The *rde-4(ne299)* mutation also has a non-identical T insertion within the same codon for Ala123 [13](Figure 3C).

**Figure 4:**
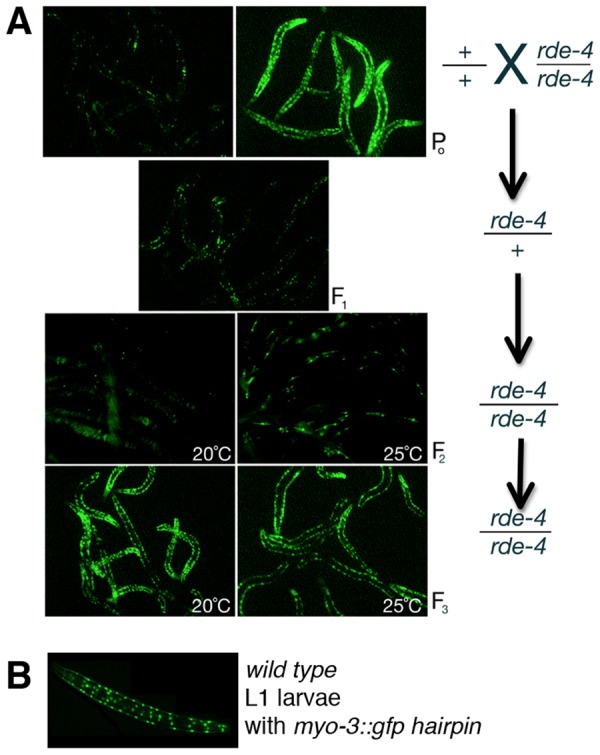
Long-lasting maternal effect are observed in *rde-4* mutants expressing dsRNA from extrachromosomal transgene arrays. All animals harbor an integrated transgene expressing GFP in muscle, as well as an extrachromosomal array expressing *gfp* dsRNA in muscle. (**A**) F2 generation *rde-4* mutants, derived from heterozygous parents, display RNAi activity due to maternal effect (third row). *rde-4* F3 animals, derived from homozygous *rde-4* parents, display RNAi defects, as evidenced by the lack of GFP reporter silencing (fourth row). Each generation of animals was derived from a series of genetic crosses, with genotypes depicted on the right. Similar results were observed for both *rde-4(ne299)* and *rde-4(ne309)* alleles. The animals depicted in (A) are adults. (**B**) Early stage wild-type L1 larvae harboring both *gfp* dsRNA trigger and *gfp* mRNA target sequences do not display RNAi. In later stages, RNAi activity in these wild-type animals becomes apparent, as depicted in (**A**), top left. 40× magnification.

Our preliminary analyses of *rde-4*(*ne309*) homozygotes revealed that the mutants were RNAi deficient in their response to ingested dsRNA, yet they displayed a systemic RNAi response to transgene-delivered dsRNA [4]. These results are inconsistent with reports of fairly strong RNAi defects in *rde-4* mutants, irrespective of dsRNA delivery method [13]. We therefore re-investigated the RNAi response to transgene-delivered dsRNA in *rde-4(ne309)* mutants. We first ensured that the strains in this study did not harbor previously described background mutations that affect RNAi activity; for example, we used strains that had been extensively outcrossed in order to remove any incidental mutations that might affect RNAi. We again used the two transgene system to target silencing of a *gfp* reporter to assess RNAi activity (as in Figure 1). In these experiments, we observed strong RNAi defects in *rde-4(ne309)* and *rde-4(ne299)* mutants, which is consistent with previous reports of strong RNAi defects in *rde-4*. By contrast, we also observed RNAi competency in *rde-4* homozygotes due to maternal effects (Figure 4A and Tables 1 and 2).

**Table 1:** Analysis of RDE-4 maternal effects.

	Crosses at 22°C					
	# F1 animals		# F2 animals		# F3 animals	
Phenotype	RNAi+ (Dim)	RNAi− (Bright)	RNAi+ (Dim)	RNAi− (Bright)	RNAi+ (Dim)	RNAi− (Bright)
cross A	47	0	14	0	4	2
cross B	68	0	15	0	6	2
cross C	73	0	31	0	17	6
	Total:				27	10

RDE-4 maternal effects are fully penetrant and expressive. Progeny animals from crosses depicted in Figure 4A were scored in each generation for RNAi activity (*see* Materials and Methods). Results from three different crosses are depicted. Three experiments were conducted at 22°C. The maternal effects were not altered by growth temperature. *rde-4/+* F1 heterozygotes displayed wild-type RNAi activity, as expected. All of the F2 animals, including *rde-4/rde-4* homozygotes, displayed wild-type RNAi activity due to RDE-4 maternal effects. Some of the F2 progeny were chosen at random, isolated, allowed to produce F3 progeny, and subsequently genotyped by DNA sequencing. Those F2 progeny that were homozygous for *rde-4* produced F3 progeny that were RNAi- at 100% penetrance and expressivity; those F2 progeny that were heterozygous or wild type produced F3 progeny that were RNAi+ at 100% penetrance and expressivity, which is in keeping with a strong RDE-4 maternal effect. The ratio of wild-type to homozygous *rde-4* F2 animals was approximately 3∶1, as predicted, and this is reflected in the RNAi activity of the F3s as well. Similar results were observed for both *rde-4(ne299)* and *rde-4(ne309)* alleles.

**Table 2:** Analysis of RDE-4 maternal effects.

	Crosses at 15°C					
	# F1 animals		# F2 animals		# F3 animals	
Phenotype	RNAi+ (Dim)	RNAi− (Bright)	RNAi+ (Dim)	RNAi− (Bright)	RNAi+ (Dim)	RNAi− (Bright)
cross A	all	0	all	0	14	4
cross B	all	0	all	0	6	3
cross C	all	0	all	0	3	1
cross D	all	0	all	0	7	1
	Total:				30	9

RDE-4 maternal effects are not affected by temperature. Four separate experiments, performed as described for Table 1, were conducted at 15°C using *rde-4(ne309).* Similar results were obtained using *rde-4(ne299)* alleles.

An RNAi response was observed in the adult somatic tissues of *rde-4* homozygotes born from a heterozygous parent, which is surprisingly long-lasting for a maternal effect (Figure 4A). Such long-lasting maternal effects have also been observed for *dcr-1* mutants [14], [15]; however, unlike *rde-4* mutants, *dcr-1* animals are sterile. There are several factors which may contribute to the perdurance of RNAi activity in *rde-4* mutants displaying such maternal effects. *C. elegans* harbors dsRNA amplification mechanisms that, in part, involve the activity of RNA-dependent RNA Polymerases and the production of secondary siRNAs [16]. Thus, any maternally deposited silencing RNAs, along with maternally deposited RDE-4 could contribute to secondary siRNA production early in development, allowing for later RNAi activity, long after the maternally deposited RDE-4 and dsRNA are eliminated. Another factor that could contribute to RNAi activity in *rde-4* adults might involve early modifications to chromatin, triggered by maternally deposited molecules, and persistence of the modifications through larval development [17], perhaps maintained by later exposure to dsRNA. Maternal effects have previously been observed for *rde-*4, yet such experiments made use of dsRNAs that were injected into heterozygotes, and these injected molecules may have persisted in the progeny animals for the duration of the experiment [18], [19]. By contrast, our experiments utilize a dual transgene system, which affords particular advantages in analyzing RDE-4 maternal effects. In this dual transgene system, the *myo-3* promoters that drive transcription of dsRNA are activated during late embryogenesis in the body wall muscles of the developing embryo [20]. Furthermore, the dsRNA sequences are expressed from a repetitive extra-chromosomal array, a DNA configuration that is normally transcriptionally silenced in the germ line [21]. The relatively late-acting nature of the promoter in this system provides additional insurance that dsRNA is not present in early developmental stages, and potentially allows time for any maternally deposited RDE-4 or dsRNA molecules to be cleared before new *gfp* dsRNA is transcribed. Indeed, 100% of the newly hatched L1 larvae from this dual transgene system do not display an RNAi phenocopy even in wild-type animals (as depicted in Figure 4B), an indication that any dsRNA molecules or chromatin modifications remaining at this stage have been eliminated or are functionally ineffective. Thus, the observation of RNAi activity in *rde-4* homozygotes at adult stage (Figure 4A) provides an indication that maternally-deposited RDE-4 persists until later stages, when it then acts upon dsRNA that is newly transcribed.

In addition to maternal effects, there are other conditions that can influence the level of RNAi activity observed in *rde-4* mutants. For example, RNAi activity has been observed in homozygous *rde-4* mutants when the dosage of dsRNA is high. Indeed, we previously observed an RNAi response in *rde-4(ne309)* mutants to injected dsRNA [4]. Earlier reports also demonstrated that *rde-4* mutants can display an RNAi response when large amounts of dsRNA are delivered by transgenes [22]. We can then infer several factors that may influence the level of dsRNA accumulation from transgenes: the copy number of dsRNA-expressing cassettes in transgene arrays, the promoter strength, and the expression status of the transgene–transgenes configured as extrachromosomal arrays are targets of epigenetic mechanisms that can lead to increased levels of transcriptional repression in a generational fashion, especially in the germ line [21]. Our earlier observations that *rde-4(ne309)* mutants were capable of mounting a systemic RNAi response to dsRNA expressed in muscle were likely influenced by the *rde-4* maternal effects, the young age of the dsRNA-expressing array (newly formed arrays are metastable with respect to transcriptional status and may be capable of higher levels of transcription than an array maintained for many generations [21]), and by the relatively low abundance of the *gfp* mRNA target used at that time [4]. Finally, environmental influences, such as temperature, have also been observed to affect the RNAi activity in wild-type as well as *rde-4* and other RNAi-related mutants [22], [23]. Taken together, these considerations highlight the possibility that dsRNA concentrations and environmental conditions might mask the presence of an underlying and unanticipated RNAi defect caused by a background mutation in a non-essential RNAi pathway component.

## Conclusions

The Dicer double-stranded RNA-specific endonuclease plays a central role in many RNA silencing pathways and is an essential gene in *Caenorhabditis elegans* and other organisms. The DCR-1 protein, in part, catalyzes the conversion of dsRNAs into small siRNAs, facilitates the formation of the RISC complex, and processes miRNA genes in *C. elegans.* DCR-1 is directed towards particular functions in the context of the associated proteins. For example, the ERI/DCR complex contains DCR-1 as well as ERI-1, RRF-3, ERI-3, and ERI-5 [24]. The ERI/DCR complex is important for the proper function of endogenous siRNAs, a pathway that is relevant to proper sperm development [25]. In another distinct DCR-1 complex can be found the RDE-1 and RDE-4 proteins. Among other activities, this complex is required for an RNAi response to experimentally-delivered dsRNA. RDE-4 is a dsRNA binding protein required for production of siRNAs from long dsRNAs [26], while the RDE-1 Argonaute protein facilitates removal of the passenger strand from the siRNA, which is necessary in order for proper function of the guide strand [27]. Mutations in genes encoding the *eri* class of DCR-1 interacting proteins enhance RNAi activity, while mutations in *rde-1* and *rde-4* strongly abrogate RNAi. Despite the important endogenous roles played by Dicer and associated proteins, most of the proteins associated with Dicer are not essential. Thus, when such genes are unknowingly mutated, there is potential for genetic complication and misinterpretation of data.

Here we report observations of *rde-4* and *rde-1* mutations that highlight the potential for maternal effects, environmental conditions, dsRNA dosage and recently derived mutations to complicate phenotypic analyses. The *rde-1(yy11)* mutation, a recently derived transposon insertion, spontaneously appeared in a strain that harbored a known RNAi-defective mutation. As a result, the effects of the background *rde-*1 mutation were at first mistakenly attributed to the known mutation. Fortunately, the *rde-1(yy11)* allele was localized to only one set of experiments, and the mutation was identified before further complications arose. The phenotypic complications related to *rde-1(yy11)* were opposite to those observed for *rde-4(ne309).* In this case, the systemic RNAi defects in a known, but not yet identified, RNAi-defective mutant were masked by maternal effects and a high ratio of dsRNA trigger molecules to mRNA target. There are many non-essential RNAi pathway mutants with RNAi defects that are more subtle than those observed in *rde-4,* and many of these mutants are dosage-sensitive with respect to the amount of dsRNA as well. Therefore, unanticipated background mutations in such genes have the potential to complicate phenotypic analysis in a similar manner.

It has become increasingly apparent in the *C. elegans* RNAi field that the possibility of background mutations must be considered. There are several steps that can be taken in order to avoid complications from unanticipated background mutations, such as performing experiments using multiple, independently derived alleles. If multiple alleles are not available, then different strains can be derived from the same mutant using different outcrossing strategies. Mutant analysis can be also compared to data derived from dsRNA-treated animals. Finally, trans-heterozygotes animals derived from crossing together strains homozygous for different alleles or for the same allele obtained from two different outcrossing strategies can be used to confirm experimental results.
